# *Cupressus sempervirens* Essential Oil: Exploring the Antibacterial Multitarget Mechanisms, Chemcomputational Toxicity Prediction, and Safety Assessment in Zebrafish Embryos

**DOI:** 10.3390/molecules27092630

**Published:** 2022-04-19

**Authors:** Sarra Akermi, Slim Smaoui, Khaoula Elhadef, Mariam Fourati, Nacim Louhichi, Moufida Chaari, Ahlem Chakchouk Mtibaa, Aissette Baanannou, Saber Masmoudi, Lotfi Mellouli

**Affiliations:** 1 Laboratory of Microbial Biotechnology and Engineering Enzymes (LMBEE), Center of Biotechnology of Sfax (CBS), University of Sfax, Road of Sidi Mansour Km 6, P.O. Box 1177, Sfax 3018, Tunisia; sarahakermi221@gmail.com (S.A.); elhadefkhawla@gmail.com (K.E.); mariamfourati@ymail.com (M.F.); moufida.chaari97@gmail.com (M.C.); ahlemchakchouk@yahoo.fr (A.C.M.); lotfi.mallouli@cbs.rnrt.tn (L.M.); 2 Laboratory of Molecular and Cellular Screening Processes, Center of Biotechnology of Sfax, University of Sfax, Road of Sidi Mansour Km 6, P.O. Box 1177, Sfax 3018, Tunisia; nacim.louhichi@gmail.com (N.L.); aissette.baanannou@yahoo.fr (A.B.); saber.masmoudi@cbs.rnrt.tn (S.M.)

**Keywords:** *Cupressus sempervirens* essential oil, antibacterial activity, membrane permeability, replication and transcription inhibition, computational toxicology, molecular docking, zebrafish

## Abstract

Nowadays, increasing interest has recently been given to the exploration of new food preservatives to avoid foodborne outbreaks or food spoilage. Likewise, new compounds that substitute the commonly used synthetic food preservatives are required to restrain the rising problem of microbial resistance. Accordingly, the present study was conducted to examine the chemical composition and the mechanism(s) of action of the *Cupressus sempervirens* essential oil (CSEO) against *Salmonella enterica* Typhimuriumand *Staphyloccocus aureus*. The gas chromatography analysis revealed α-pinene (38.47%) and δ-3-carene (25.14%) are the major components of the CSEO. By using computational methods, such as quantitative structure–activity relationship (QSAR), we revealed that many CSEO components had no toxic effects. Moreover, findings indicated that α-pinene, δ-3-carene and borneol, a minor compound of CSEO, could inhibit the AcrB-TolC and MepR efflux pump activity of *S. enterica* Typhimurium and *S. aureus*, respectively. In addition, our molecular docking predictions indicated the high affinity of these three compounds with active sites of bacterial DNA and RNA polymerases, pointing to plausible impairments of the pathogenic bacteria cell replication processes. As well, the safety profile was developed through the zebrafish model. The in vivo toxicological evaluation of (CSEO) exhibited a concentration-dependent manner, with a lethal concentration (LC_50_) equal to 6.6 µg/mL.

## 1. Introduction

It is clear that the long-term use of antibiotics has provoked the mass production of genetically resistant bacteria [1]. As a result, some pathogenic bacteria have become resistant to entire antibiotics classes [1,2,3]. For instance, some bacterial species usually susceptible to carbapenems and colistin, such as *Enterobacteriaceae*, *P. aeruginosa* and *K. pneumoniae*, have gained the ability to hydrolyze β-lactams and make them highly resistant to most β-lactam antibiotics [1]. In addition, methicillin-resistant *Staphylococcus aureus* (MRSA) and extended-spectrum β-lactamase producers are resistant not only to methicillin and cephalosporin, but to tetracycline, aminoglycosides, macrolides, and chloramphenicol [4].

By virtue of the extensive bacterial resistance to numerous drugs and antibiotics, bacterial infections have become great health challenges, creating expanded concern in the search and the development of new antimicrobial agents. In this regard, during the past 20 years, the number of new drugs that have reached the marketplace has significantly decreased and the number of new antibiotics approved for marketing is in continuous decline. Thus, there is an urgent need for new discovery strategies to control antibiotic-resistant bacteria. One of the main approaches is the identification and exploitation of new targets in pathogens. Recently, with the dramatic reduction in the cost of bacterial genome sequencing and the development and evolution of bioinformatic tools, it has become possible to compare several bacterial genome sequences including those of pathogenic bacteria, to understand the interactions between targets and active compounds and consequently, to predict novel therapeutic targets against pathogenic microorganisms.

One of the easily manageable origins of such compounds are medicinal plants that offer a wide kind of phytochemicals with antimicrobial activity [5,6]. Plants and their derivatives, including essential oils (EO), have presented a broad range of secondary metabolites that are commonly reported to prevent or delay the growth of bacteria, yeasts, and molds [7,8,9]. In this respect, the antibacterial mechanism of EOs could be related to the phenolic constituents and their interaction with minor constituents [10,11,12]. However, the compounds’ hydrophobicity, presented in EOs, permitted them to transfer throughout the cell wall and cytoplasmic membrane, perturb the structure of their different layers of polysaccharides, fatty acids, and phospholipids, and, eventually, permeabilize them [10,11]. Additionally, EOs can inhibit diverse enzyme systems covering the enzymes responsible for managing energy and synthesis of structural components [12]).

Containing twelve plant species, the genus *Cupressus* is distributed in different parts of the world, such as the Mediterranean regions [13]. *Cupressus sempervirens* is the sole species of this genus indigenous from Tunisia [14] and has been customarily utilized for influenza and rheumatism treatments, as an antiseptic, and against the inflammation, colds, curing diabetes [15]. From a chemical point of view, previous studies conducted on *C. sempervirens* EO (CSEO) have revealed that it contains various bioactive substances such as α-pinene [14,15], β-caryophyllene, and germacrene D [15], which are described to have considerable antimicrobial potentials [15,16,17].

On the other hand, the toxicological profiles of the majority of medicinal plant EOs have not been greatly elucidated. In this way, toxicity challenging in a varied range of in vitro studies using animal models is crucial and comprises experimental screening methods for determining the safety profile of EOs. By way of illustration, zebrafish embryos are helpful for assessing vertebrate development of endpoint morphological changes in toxicity studies [18,19]. Indeed, each growth stage (from fertilization, embryogenesis, and organogenesis to larva hatching) matches other higher vertebrates’ embryogenesis, including humans [19,20,21].

The objective of our paper was to highlight the updated focus on the (CSEO) application in food preservation. By skillfully using in silico and software tools, here, molecular docking interactions of all (CSEO) compounds with bacterial DNA and RNA polymerases and DNA topoisomerase II (DNA gyrase) of the two pathogenic bacteria *Staphylococcus aureus* and *Salmonella enterica* Typhimurium, as well as molecular docking interactions of the major (CSEO) constituents with the cell membrane of these two pathogenic bacteria, were investigated. In addition, the in vivo innocuity of acute exposure of the efficient concentration of *C. sempervirens* EO was explored.

## 2. Results and Discussion

### 2.1. Chemical Composition Analysis of CSEO

(CSEO) GC-MS analysis exhibited 27 different components (Table 1). The main components were α-pinene, the most abundant compound (38.47%), δ-3-carene (25.14%), D-limonene (5.84%), and citronellal (5.33%). Additionally, four components were present in more than 2% of the (CSEO), which are *α*-terpinyl acetate (2.82%), β-myrcene (2.78%), cedrol (2.24%), and β-pinene (2.04%).

*α*-Pinene [2,6,6,-trimethylbicyclo(3.1.1)-2-hept-2-ene] is a natural and active monoterpene that is used in flavorings, fragrances, insecticides, fine chemicals, and pharmaceuticals [22]. Several studies have attributed interesting biological activities to *α*-pinene, including antimicrobial [23], hypertensive [24], antinociceptive [25], and anti-inflammatory [26]. In addition, the US Food and Drug Administration [27] approved this compound as a food additive generally recognized as safe. δ-3-carene, a bicyclic monoterpene, is widely known for its antimicrobial activity, notably against *Aspergillus* and *Candida* species [28], antitussive and expectorant properties [29], and its activity against acute inflammation [30]. D-limonene, a simple monocyclic monoterpene, has been used as a flavor and fragrance additive in cleaning and cosmetic products, food, beverages, and pharmaceuticals [31] and has demonstrated potential chemo-preventive and anticancer activity in preclinical and clinical studies [32]. The monoterpenoid citronellal presents many interesting clinical activities like its central nervous system depressant and anticonvulsant [33] and its potential benefit in managing inflammatory disorders and correlated damage caused by oxidant agents [34].

In our case, the major constituents of (CSEO) were α-pinene, δ-3-carene, D-limonene, and citronellal. Nevertheless, it should be noted that the essential oil composition of plants is closely related to the location of the plant and the method used to extract and isolate essential oils. In fact [35], have studied the aerial parts of the same plant *Cupressus sempervirens* collected from Makther in Tunisia, using the same hydrodistillation technique, obtained a significant variation in (CSEO) composition and the major compounds were α-pinene (31.61%), α-cedrol (13.50%), δ-3-carene (9.50%) and germacrene D (8%). This difference in both composition and compounds percentage of (CSEO) can be explained by the fact that the region of Sfax, Tunisia, is characterized by semi-arid climatic conditions with an annual average precipitation of 230 mm, while Makther, Tunisia, is known for its Mediterranean climate and about 450 mm of precipitation falls annually in this region. World widely, the literature revealed wide variations in the (CSEO) collected from different locations [14,17].

### 2.2. Antibacterial Activity

(CSEO) has been screened for its antibacterial activity against four bacterial strains (two Gram-positive and two Gram-negative bacteria). The antibacterial activity was assessed by evaluation of the growth inhibition zones and the determination of MIC values. As compiled in Table 2, (CSEO) showed antibacterial activity against the four tested strains with inhibition zones of 21 and 15 mm against Gram-positive and Gram-negative bacteria, respectively. However, it should be noted that the monoterpene family is known by its antibacterial activity [36], and in our case, this family represented a high level (74.78%) of the (CSEO) composition with α-pinene (38.47%), δ-3-carene (25.14%), D-limonene (5.84%), and citronellal (5.33%). The obtained MIC values (Table 2), indicated that (CSEO) was most effective against Gram-positive bacteria (MICs = 6.25 µg/mL) than Gram-negative bacteria (MICs = 12.5 µg/mL).

These data were in concordance with previous findings and it has been reported that Gram-positive bacteria were more sensitive to plant essential oils than Gram-negative bacteria. The resistance of these latter bacteria to plant essential oils was attributed to the presence of external lipopolysaccharide surrounding the peptidoglycan cell wall, which acts as a hydrophobic barrier to essential oils [37]. Concerning the antibiotic gentamicin used as standard, the antibacterial activity was higher against Gram-negative bacteria than Gram-positive bacteria with an inhibition zone of 25 mm and an MIC value of 2.5 µg/mL against Gram-negative bacteria, and an inhibition zone of 20 mm and an MIC value of 12.5 µg/mL against Gram-positive bacteria (Table 2). It should be mentioned that gentamicin is an aminoglycoside bactericidal and is a broad-spectrum antibiotic active against a wide range of bacterial infections; mostly Gram-negative bacteria [38].

### 2.3. Compounds Toxicity Evaluation by In Silico Tools

#### 2.3.1. Computational COMPOUND Toxicity Prediction by VEGA HUB Software

By using VEGA HUB software: the QSAR (quantitative structure–activity relationship) approach, the toxicity of some selected food preservatives recommended by FDA (Food and Drug Administration), approved antibiotics: rifamycin SV and ciprofloxacin, efflux pump inhibitors (EPIs): cathinone and thioridazine, and (CSEO) compounds were elucidated (Table 3).

The selected antibiotics are predicted to be toxic in different assays. In this regard, rifamycin SV and ciprofloxacin are found to be mutagenic in the mutagenicity test/model (Ames test) and predicted to be toxic in the developmental toxicity model. These two antibiotics also produce active genotoxicity signals in the in vitro micronucleus activity model. Similar trends have been observed in recommended food preservatives. The latter showed some toxicity measurements and none of the selected preservatives were detected to be nontoxic (Table 4). A previous study conducted by Damayanti et al. (2015) evaluated the toxicity of some food preservatives in silico using Toxtree and OECD QSAR Toolbox software [39]. These authors reported that ascorbic acid is slightly toxic; butylhydroxyanisole (BHA) was predicted to be moderately toxic, carcinogen, and could engender reproduction toxicity; and citric acid was demonstrated to be slightly toxic and carcinogenic.

Additionally, EPIs were predicted to cause reproductive/developmental toxicity and produce genotoxic signals. For instance, cathinone’s toxicity was previously evaluated in vitro and experiments confirmed that this EPI is hepatotoxic and causes huge damage in the liver [40]. Similarly, thioridazine has been associated with liver toxicity [41]. Therefore, these molecules are quite unsafe and can be harmful to human health.

Interestingly, many (CSEO) components such as β-terpinene, α-fenchene, sabinene, δ-3-carene, citronellal, borneol, β-citronellol, α-fenchyl acetate, camphene and α-terpinyl acetate showed no toxicity effect. This predicts that these molecules can be safe antimicrobial agents, economically low-cost choice as compared to synthetic antibiotics and could also be used as bio-preservative agents in a view to extend the shelf life of stored products, enhance the nutritional quality and guarantee the safety of food for future consumption.

#### 2.3.2. Rodent Oral Toxicity and Cytotoxicity of (CSEO) Compounds Predicted by PROTOX II Tool

The oral toxicity of (CSEO) compounds in rodents was predicted using the webserver PROTOX II. This tool divided the compounds into different classes based on their lethal dose upon swallowing [42,43]. None of the (CSEO) compounds were found to cause cytotoxicity. In addition, except for P-cymene, LD_50_ values of the 27 (CSEO) compounds and the two controls did not reveal any fatal or toxic molecule which belongs to Class 1, 2, and 3. Remarkably, the two (CSEO) major compounds, α-pinene and δ-3-carene, were predicted to belong to Class 5, which means that they can be harmful if swallowed (2000 < LD50 ≤ 5000). Further, tested food preservatives were predicted to belong to class 3 and 4, meaning that these substances could be toxic if swallowed (50 < LD50 ≤ 300) and harmful if swallowed (300 < LD50 ≤ 2000), respectively, except L-ascorbic acid, which was categorized into class 5 (Table 5). Finally, cathinone and thioridazine were predicted to belong to class 4 (harmful if swallowed (300 < LD50 ≤ 2000). The same model was used to evaluate the toxicity of chlorogenic acid in order to use it as a promising efflux pump inhibitor against AcrB protein of *E. coli* TG1. It was demonstrated to have a high LD50 value indicating that it is nontoxic [44]. Results of toxicity evaluation by the use of in silico tools confirmed that (CSEO) compounds could be safely used as antibacterial agents and biopreservatives as compared to synthetic food preservatives and FDA-approved antibiotics.

### 2.4. In Vivo Toxicity Assessment Using Zebrafish Model 

**Figure 1:**
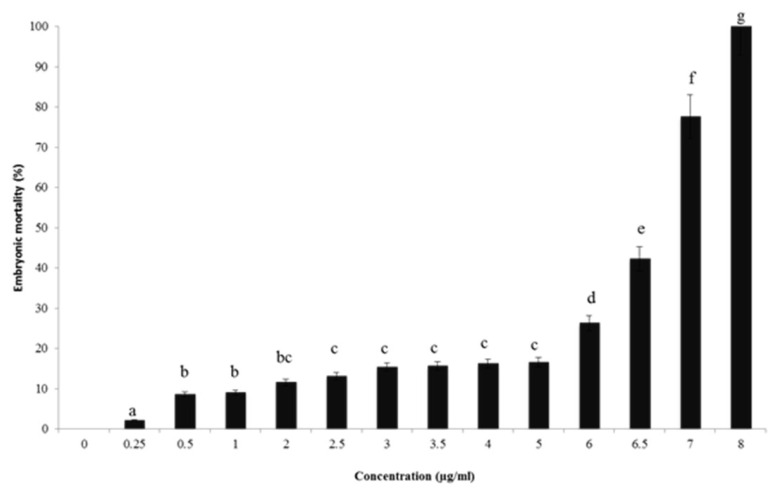
The embryotoxicity of different concentrations of (CSEO) in embryonic zebrafish mortality. All tests were performed in triplicate; Values with a different letter (a–g) are significantly different (*p* < 0.05).

By using the zebrafish model in toxicity screening, findings showed that the use of DMSO at 0.1%, used as a positive control, did not display any toxicological effect on zebrafish embryonic development (Figure 1). This result came to approve previous studies achieved by Hoyberghs et al. (2020) [45] and Thitinarongwate et al. (2021) [21]. For concentrations superior to 6 µg/mL, a significant (*p* < 0.05) increase in mortality rate in zebrafish embryos was detected. In addition, no viable zebrafish embryos were observed for the groups treated with 8 μg/mL of (CSEO); therefore, (CSEO) toxicity is a concentration-dependent effect. On the other hand, the LC_50_ obtained in the present study was 6.6 μg/mL. It should be noted that we studied the toxicity of all 27 components of (CSEO) separately by using the Vega QSAR model and, according to Table 3, we have demonstrated that some components are totally safe and others possess a limit of toxicity. Among these later, α-pinene developed toxicity only in the developmental toxicity model (CAESAR) 2.1.7 out of nine toxicity measurements. Concerning the in vivo study (LC50), we used the whole (CSEO), and consequently, we must find a certain limit of toxicity.

Similar assays were conducted using the zebrafish model to assess different EOs’ toxicities. Thitinarongwate et al. (2021) evaluated the toxic effects of *Zingiber ottensii* Valeton (ZO) EO [21]. These authors concluded that the LC_50_ value was equal to1.003 µg/mL, meaning that ZO EO showed more toxic effects on zebrafish embryos as compared to the query EO.

### 2.5. Antibacterial Mechanisms of (CSEO)

#### 2.5.1. Alteration of Bacterial Cell Permeability: Inhibition of Efflux Pumps by (CSEO)

To better understand the mechanism of membrane permeability alteration by inhbiting efflux pumps against *S. enterica* Typhimurium and *S. aureus*, two major foodborne pathogen bacteria, docking studies of all (CSEO) components were carried out. In this approach, the AcrB efflux pump protein model of *S. enterica* Typhimurium and the cristallographic structure of MepR of *S. aureus* (PDB ID: 3ECO) were established. Homology modeling results displayed that AcrB model identity was 94.47% with a QMEAN value equal to −3.04 and Ramachandran Plot values of favored regions and allowed regions were >90%.

It should be noted that the AcrAB-TolC is the major RND (resistance–nodulation–division) efflux system providing the *S. enterica* Typhimurium resistance to many antibiotics [46]. The AcrAB-TolC system is specifically formed by the AcrB efflux pump associated with an outer membrane protein (TolC). This complex pump out therapeutic molecules accumulated in bacterial periplasmic space after binding to them, resulting to a broad substrate specificity against several classes of antibiotics (phenicols, cyclins, β-lactams, fluoroquinolones) [47]. On the other hand, MepR is a multidrug binding transcription regulator that represses the expression and the activity of the multidrug efflux pump MepA of *S. aureus* [48]. MepA is a transporter that belongs to the MATE family (multidrug and toxic compound extrusion). This efflux pump is able to engender antibiotic resistance in *S. aureus* by extruding hydrophilic antibiotics such as fluoroquinolones and aminoglycosides [49]. MepR was chosen for molecular docking to understand the mechanism of MepA efflux pump inhibition.

Docking results displayed that the two major compounds of (CSEO), α-pinene and δ-3-carene showed free energy of binding at −6.7 Kcal/mol and −6.4 Kcal/mol, respectively (Table 6). These later free energies of binding are better than cathinone. This alkaloid, basically used as a dopamine stimulator in the central nervous system (amphetamine), showed a free energy of binding equal to −4.8 Kcal/mol and employed against *S. enterica* Typhimurium by inhibiting the function of AcrABTolC efflux pump [50,51]. Moreover, it was reported that the repression of AcrB efflux function can induce loss of virulence in S. *enterica* Typhimurium by the reduction of bacterial factors involved during infection, leading to an alteration of noxious molecules retention inside the bacterium [46].

Previous studies reported that the α-pinene detected in *Alpinia Katsumadai* seeds EO can inhibit the activity of Gram-negative efflux pumps [52]. It also showed an inhibitory effect against *Campylobater jejuni* (Gram negative bacterium) by reducing CmeABC and Cj1687 efflux pumps activities in order to increase bacterial susceptibility to ciprofloxacin, erythromycin, and triclosan [52]. Another study confirmed the existence of a synergitic effect between different monoterpenes hydrocarbons compounds present in *Citrus aurantium* L EO such as pinene, δ-3-carene and D-limonene in a view to act as bio-enhancer of antibiotics and to limit the emergence of drug-resistant infections [53].

However, it’s important to mention also that the borneol, an oxygenated monoterpene, showed an important inhibitory effect against AcrB efflux pump of *S.enterica* Typhimurium with the best free energy of binding value (−7.9 Kcal/mol) (Table 6). A previous study indicated that *Thymus* species such as *(T. broussonetii*, *T. marocanus*, *T*. *riatarum*) were proven to inhibit the AcrAB-TolC efflux system of some *Enterobacteriaceae* strains due to their high content in monoterpenes (carvacrol and borneol) which could act as efflux pump substrates and to disrupt bacterial membranes [54].

On the other hand, regarding anti-*S. aureus*, α-pinene and δ-3-carene revealed a great inhibitory effect on MepR, the transcription regulator of the MepA efflux pump, with free energies of binding equal to −6.5 Kcal/mol and −6.2 Kcal/mol, respectively (Table 6). Similar free energy of binding was detected for thioridazine (−6.7 Kcal/mol). This molecule had an inhibitory effect on efflux pumps of methicillin-resistant *Staphylococcus aureus* (MRSA) [55,56,57,58]. The previous study conducted by De Medeiros et al. (2017) showed that the presence of α-pinene, as a main component of *Croton growioides* EO, may modulate and reduce the activity of another efflux pump (NorA) of *S. aureus* in order to overcome bacterial resistance to antibiotics [59].

Remarkably, borneol showed an interesting affinity towards MepR (−7.7 Kcal/mol), as indicated in Table 6. To the best of our knowledge, none of the previous studies has reported the inhibitory effect of borneol on the MepR or MepA efflux pump. However, it has been generally described that some oxygenated monoterpenes such as nerol and 3,7-dimethyl-1-octanol were able to potentiate the antibiotic activity of norfloxacin and act as efflux pump inhibitors of NorA of *S. aureus* [60]. These in silico results confirmed the fact that (CSEO) compounds have a potential inhibitory effect which consists of the disruption of bacterial membrane permeability by inhibiting the function of efflux pumps.

**Figure 2:**
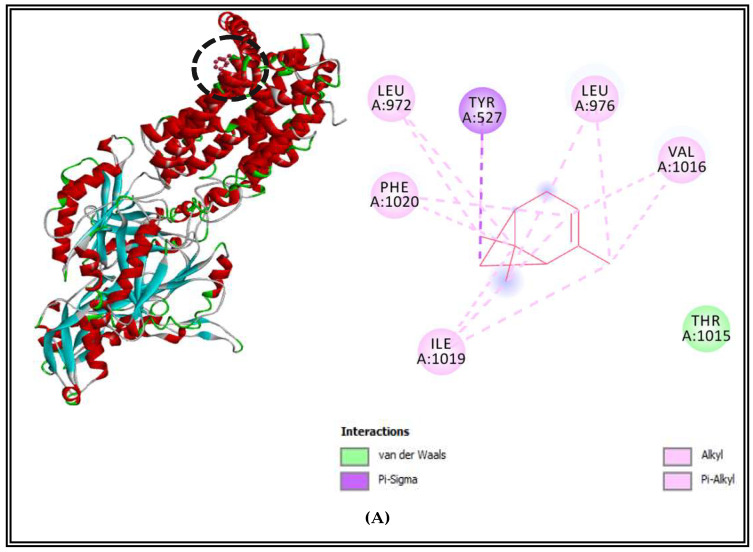

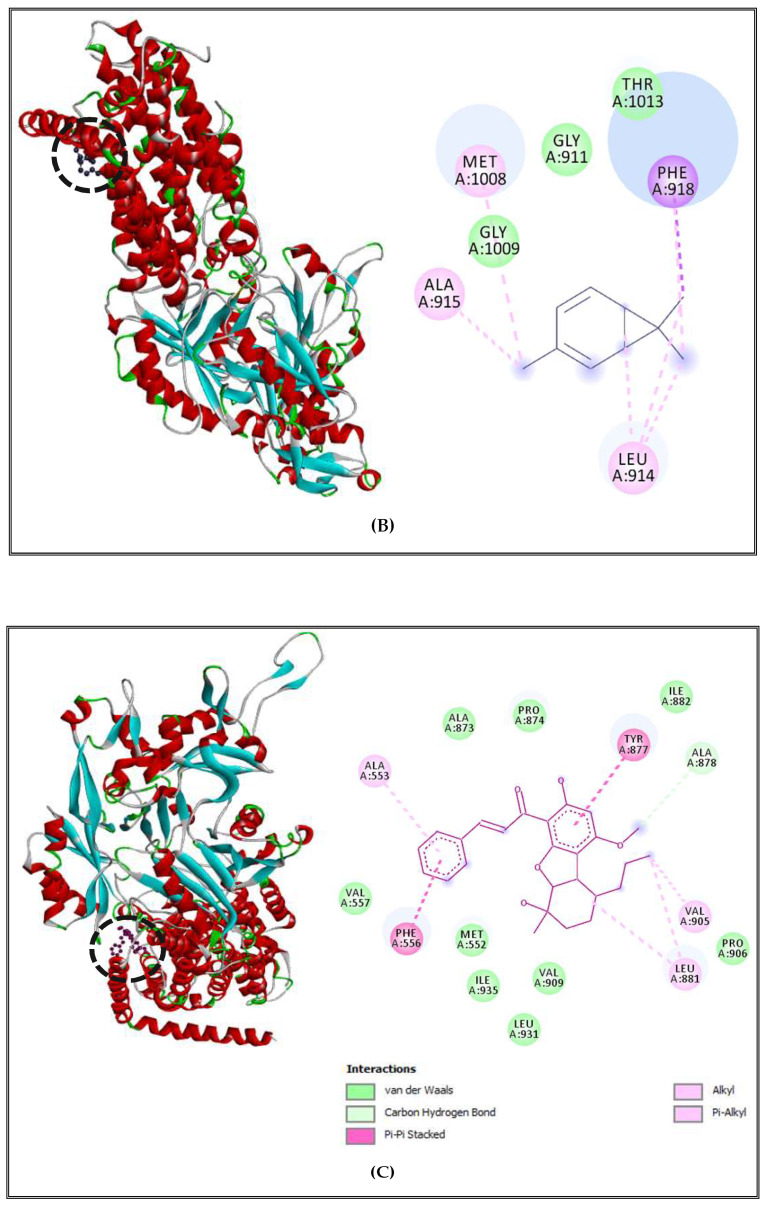
AcrB efflux pump receptor of *S. enterica* Typhimurium complexed with α-pinene (**A**), δ-3-carene (**B**), and borneol (**C**).

**Figure 3:**
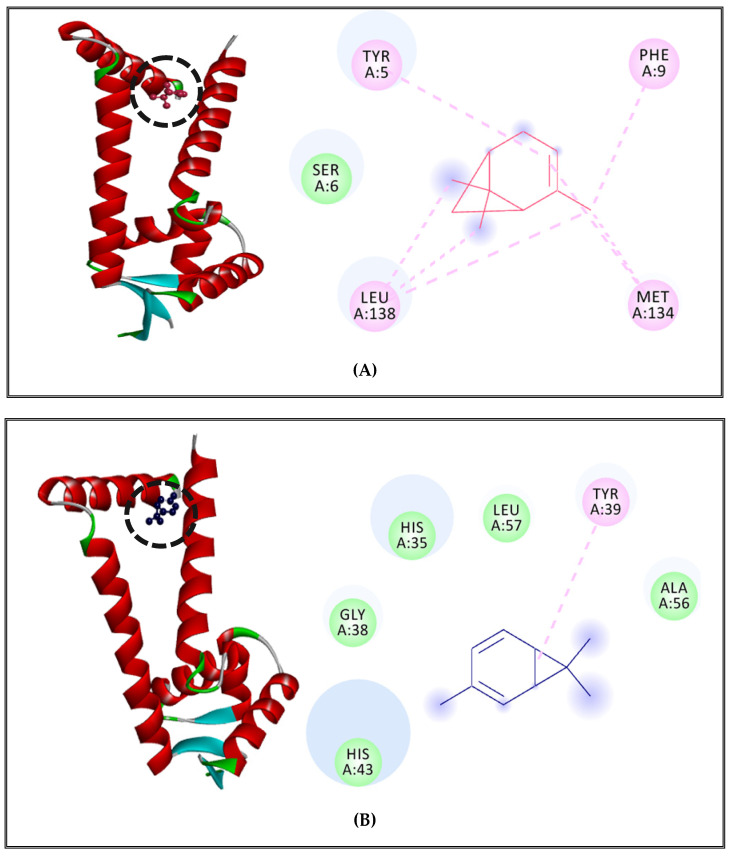

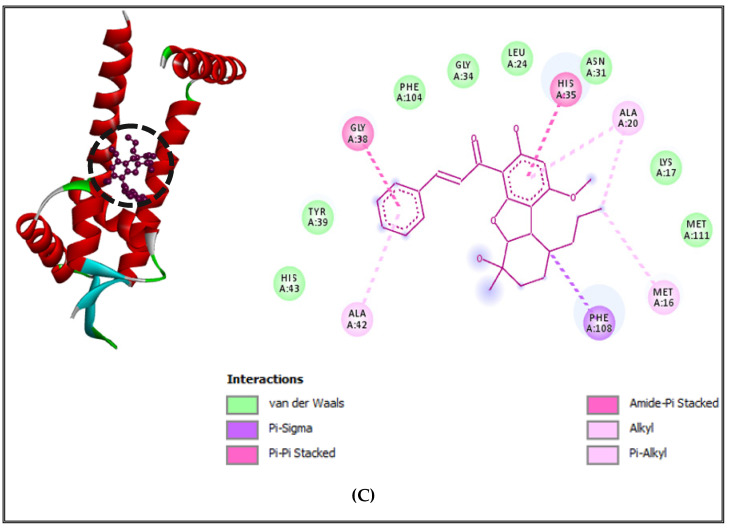
MepR receptor of *S. aureus* complexed with α-pinene (**A**), δ-3-carene (**B**), and borneol (**C**).

The results of the interaction profiles between (CSEO) major compounds and the selected efflux pumps proteins of the two pathogens bacteria are represented in Figure 2 and Figure 3. Results indicated that AcrB efflux pump receptor of *S. enterica* Typhimurium complexed with α-pinene showed Pi-alkyl and alkyl-type interactions with LEU972, LEU976, PHE1020, VAL1016, ILE 1019, and Van der Waals interaction with THR1015 (Figure 2A). The complex of δ-3-carene with AcrB efflux pump receptor presented alkyl and Pi-alkyl interactions with MET1008, ALA915, LEU914, and Van Der Waals interactions with GLY1009, GLY911, and THR1013 (Figure 2B). Additionally, borneol complexed with the AcrB efflux pump of *S. enterica* Typhimurium and displayed the existence of 5 types of interactions with nonpolar amino acids which have hydrophobic character. It showed Pi-alkyl and alkyl interactions with ALA553, LEU88, VAL905; also Van der Waals interactions with ALA873, PRO874, ILE382, Val909, LEU931, PRO906, ILE935, MET552, VAL557; one conventional hydrogen bond with ALA878, and Pi-stacked interactions with a polar amino acid TYR 877 and nonpolar aromatic amino acid PHE 556 (Figure 2C).

MepR transcription regulator of the MepA efflux pump complexed with α-pinene showed Pi-alkyl and alkyl interactions with TYR5, PHE9, LEU138, MET134 and a Van der Waals interaction with SER6 (Figure 3A). The complex of δ-3-carene with the MepR receptor showed Pi-alkyl interaction with TYR39 and Van der Waals interactions with HIS35, LEU57, GLY38,ALA56, and HIS43 (Figure 3B). Finally, borneol complexed with MepR, indicating the existence of 6 types of interactions as compared to both major compounds, which showed just 2 types of interactions. It showed the existence of alkyl and Pi-alkyl interactions with the nonpolar amino acids, which have hydrophobic characteristics such as MET16, ALA42, ALA20; Van der Waals interactions with polar amino acids such as HIS43, TYR39,ASN31, LYS17 and nonpolar amino acids such as GLY34, PHE104, LEU24, and MET111; Pi-sigma with nonpolar aromatic amino acid PHE108, Pi–Pi stacked and amide stacked with nonpolar amino acid GLY38 and a polar amino acid HIS35 (Figure 3C).

The docking scores and (CSEO) compounds’ interaction profiles with target proteins confirmed that α-pinene, δ-3-carene, and borneol can bind to multiple targets involved in the efflux pump inhibition process and have a potential to block the AcrAB-TolC pump of *S. enterica* Typhimurium and the MepR transcription regulator of MepA of *S. aureus*. It might also be employed in other efflux pumps mechanisms.

These findings support the fact that monoterpenes present naturally in EO have the ability to inhibit efflux pumps mechanisms due to their hydrophobic character [61]. Monoterpenes could alter the membrane’s permeability by moving to the bacterial membrane and interacting with polysaccharides, phospholipids, and fatty acids [62].

It is very interesting to pass from in silico methods to in vitro analysis, and it is already among our perspectives. Therefore, we project in subsequent work to perform further in vitro assays either by analyzing efflux of k+ and extracellular nucleotide leakage or by observing microscopic changes in cell structure and simulation experiments of artificial cell membranes.

#### 2.5.2. Interactions between CSEO Molecules and Bacterial Topoisomerase II, DNA and RNA Polymerases

In order to understand the mechanism of the interactions between the (CSEO) compounds and pathogenic bacteria, some bacterial receptors were selected as possible targets. In this part, we investigated the inhibitory effect of (CSEO) on Topoisomerase II (DNA gyrase) and DNA and RNA polymerases of two foodborne bacteria *Staphylococcus aureus* and *Salmonella enterica* Typhimurium. Molecular homology results of the target- templates, their identity percentages, and their corresponding Ramachandran plot and QMEAN values are presented in Table A1. Templates can be used for homology modeling when their identities (%) are higher than 30% [63]. Hence, the identities of the selected templates were over 30% and Ramachandran Plot values of favored regions and allowed regions were, together, over 90%, therefore, predicted models can be used for molecular docking simulation [64].

Nevertheless, it is important to mention that DNA and RNA polymerases are two crucial enzymes which are playing an imperative role in DNA replication, transcription and translation as well as influencing the nucleic acid formation in bacterial cells [65]. Without forgetting that topoisomerase II (DNA gyrase) is also another pivotal enzyme in DNA transcription and translation. This enzyme can catalyze the unwinding of supercoiled DNA strands and is implicated in DNA replication and transcription processes [66]. For better understanding, we choose to perform molecular docking to simulate the binding mode and to estimate the inhibitory potential of (CSEO) on pathogenic bacteria by predicting the interaction energies between each compound and Topoisomerase II, DNA and RNA polymerases.

The main components of the tested (CSEO) were complexed with different bacterial receptors, and the results of binding affinity were elucidated in Table 7. Docking results showed that the two major compounds of (CSEO), α-pinene and δ-3-carene, showed a good inhibitory effect on Topoisomerase II, RNA polymerase, and DNA polymerase of both analyzed bacteria.

Previous studies reported that α-pinene has an important antibacterial activity against several Gram-negative and Gram-positive bacterial strains and activity against methicillin-resistant *staphylococcus aureus* (MRSA) [67,68]. It was also disclosed that it can cause damage to DNA and to bacterial membranes by increasing its permeability [69]. Moreover, α-pinene present in rockrose essential oil (39.25%) was employed to develop films used in food packaging in order to extend product shelf-life [70]. On the other hand, δ-3-carene could interrupt biofilm formation and cause damage to bacterial biosynthetic pathways [71]. Generally, EOs rich in α-pinene and δ-3-carene exhibit a stronger antimicrobial activity and could be employed as a good source of natural food preservatives [72].

At a percentage equal to 1.37, borneol showed the lowest free energy of binding (Kcal/mol) and the best inhibitory potential with topoisomerase II, DNA and RNA polymerase in both analyzed bacteria (Table 7). Previous studies have revealed the important antibacterial activity of borneol [73,74]. It was used as an antibacterial agent, showing excellent bactericidal activity via membrane disruption mechanism, especially against MRSA [75]. In addition, in other studies, borneol was reported to have anti-adhesion effects by minimizing bacterial attachment and biofilm formation [76,77]. This alcohol monoterpene exhibited great antibacterial activity by causing damage and impairment to the bacterial cell membrane. It presented broad-spectrum activity against both Gram-positive and Gram-negative bacteria and it was encapsulated and used as general surface disinfectants and as antiseptics for food preservation due to its effectiveness and safety [78].

The inhibition of bacterial DNA replication mechanism by the use of EOs was previously confirmed by studies reported by Dai and al. (2020) [79]. These authors analyzed the inhibitory effect of *Litsea cubeba* EO on topoisomerase, DNA and RNA polymerases of *E.coli*. De Souza-Moura et al. (2020) studied the antibacterial activity of *Siparuna guianensis* EO and revealed the inhibitory effect of germacrene B against bacterial DNA and RNA polymerases of *E.coli, P. aeruginosa, S. aureus*, and *S. pyogenes* [80]. Therefore, these findings revealed that (CSEO) has a potential inhibitory effect on pathogenic bacteria based on the inhibition of genetic material synthesis.

Interactions details of α-pinene, δ-3-carene, and borneol with the active sites of selected bacterial targets are summarized in (Table 8).

**Figure 4:**
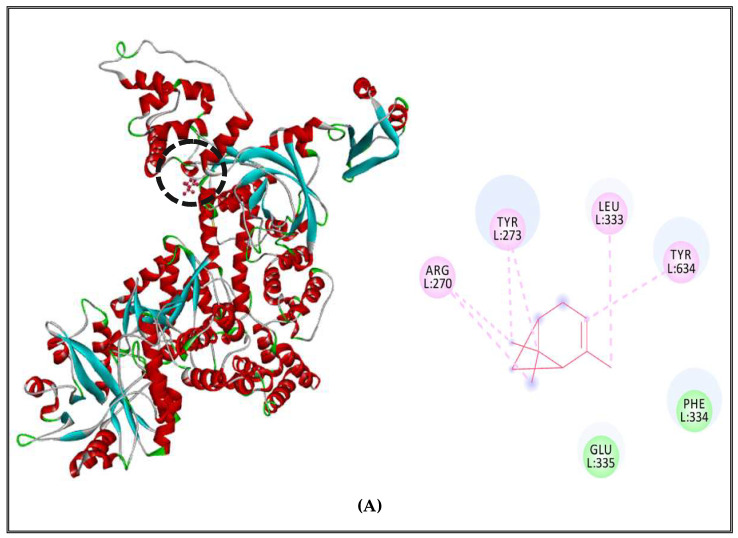

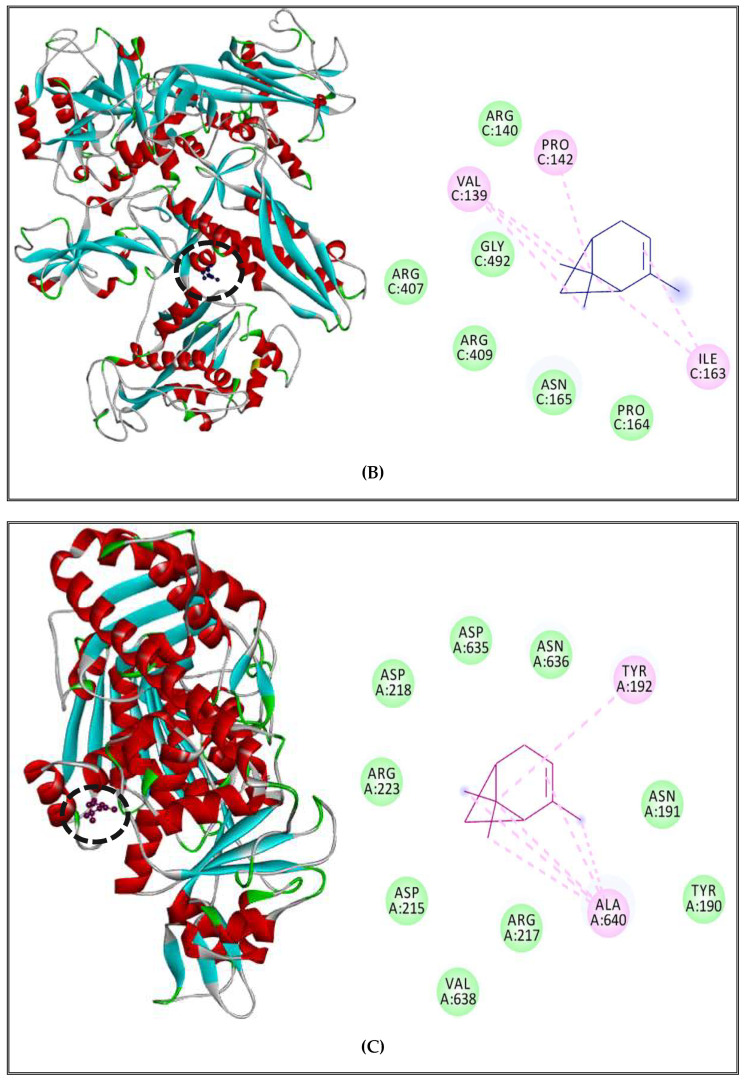
α-Pinene complexed with DNA polymerase enzyme (**A**), RNA polymerase (**B**), topoisomerase II (**C**) of *S. aureus*.

**Figure 5:**
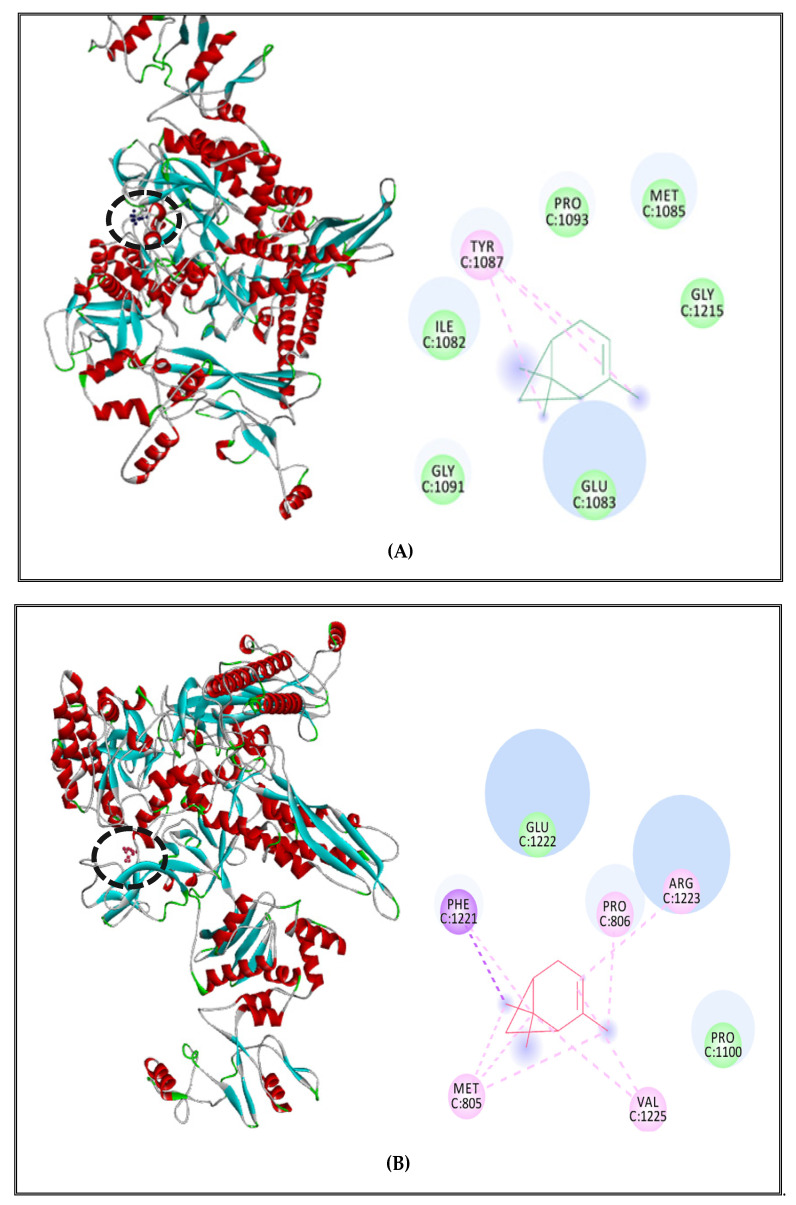

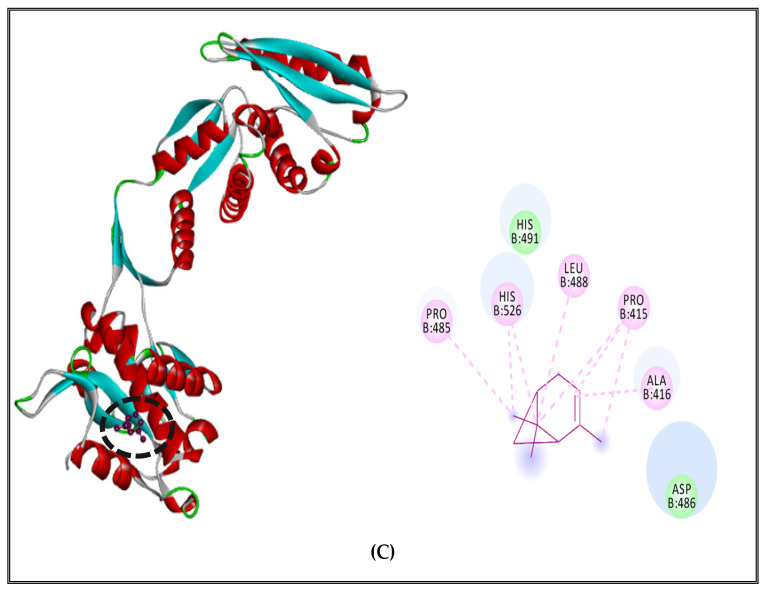
α-Pinene complexed with DNA polymerase enzyme (**A**), RNA polymerase (**B**), topoisomerase II (**C**) of *S. enterica* Typhimurium.

The results of interaction profiles between α-pinene and topoisomerase II, DNA and RNA polymerases of *S. aureus* and *S*. Typhimurium are presented in Figure 4 and Figure 5**.** α-pinene made a complex with DNA polymerase receptor via alkyl and Pi-alkyl interactions with ARG270, TYR273, LEU333, TYR634 and Van der Waals interactions with GLU335 and PHE334 (Figure 4A). In addition, RNA polymerase complexed with α-pinene showed Alkyl interactions with PRO142, VAL139, ILE163 and Van der Waals interactions with ARG140, ARG407, GLY492, ARG409, ASN165 and PRO164 (Figure 4B). Likewise, it interacted with Topoisomerase II via Alkyl and Pi-alkyl interactions with ALA640, TYR192 and it made Van derWaals interactions with ASN636, ASP635,ASP218, ARG223, ASP215, VAL638, ARG217, TYR 190 and ASN191 (Figure 4C).

α-Pinene complex with *S. Typhimurium* DNA polymerase showed interactions with active site amino acids and the ligand: Pi-alkyl interaction with TYR1087 and Van der Waals interactions with GLY1091, GLU1083, ILE1082, PRO1093, MET1085, and GLY1215 (Figure 5A)**.** For the RNA polymerase, we found alkyl and Pi-alkyl interactions with PRO806, ARG1223, VAL1225, MET805, and only two Van der Waals interactions with PRO1100 and GLU1222 (Figure 5B)**.** Concerning topoisomerase II, α-pinene presented alkyl and Pi-alkyl interactions with PRO485, HIS526, LEU488, PRO415, ALA416, and Van der Waals with HIS491 and ASP486 (Figure 5C).

**Figure 6:**
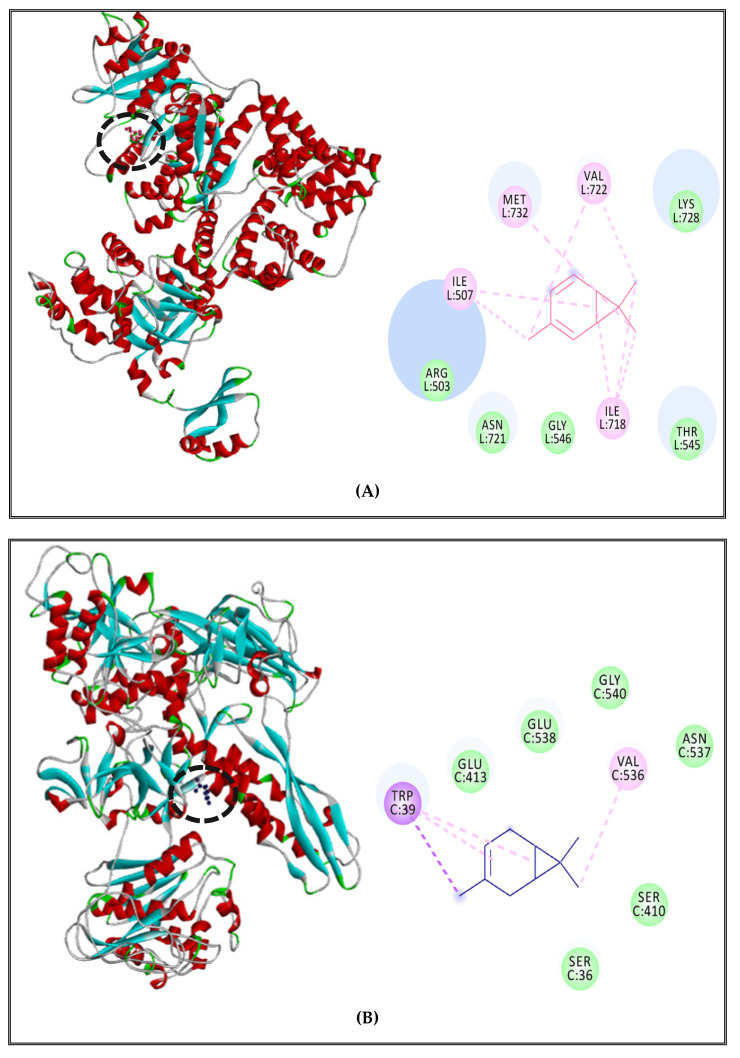

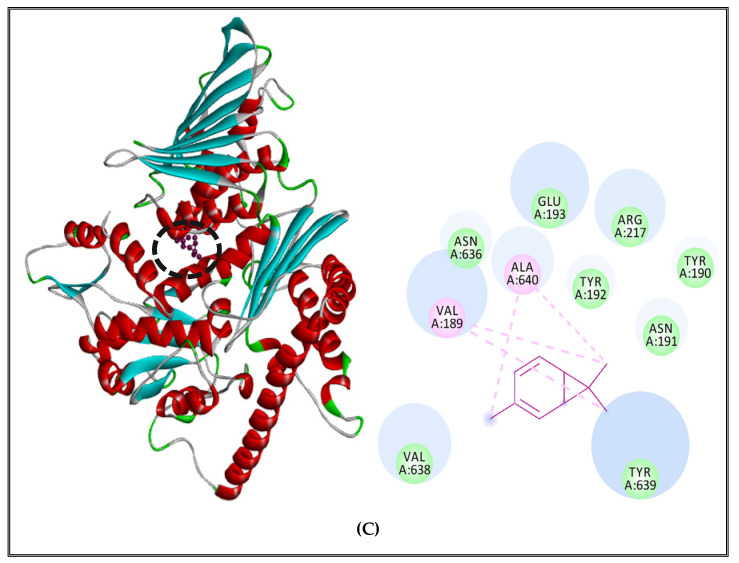
δ-3-Carene complexed with DNA polymerase enzyme (**A**), RNA polymerase (**B**), topoisomerase II (**C**) of *S. aureus*.

**Figure 7:**
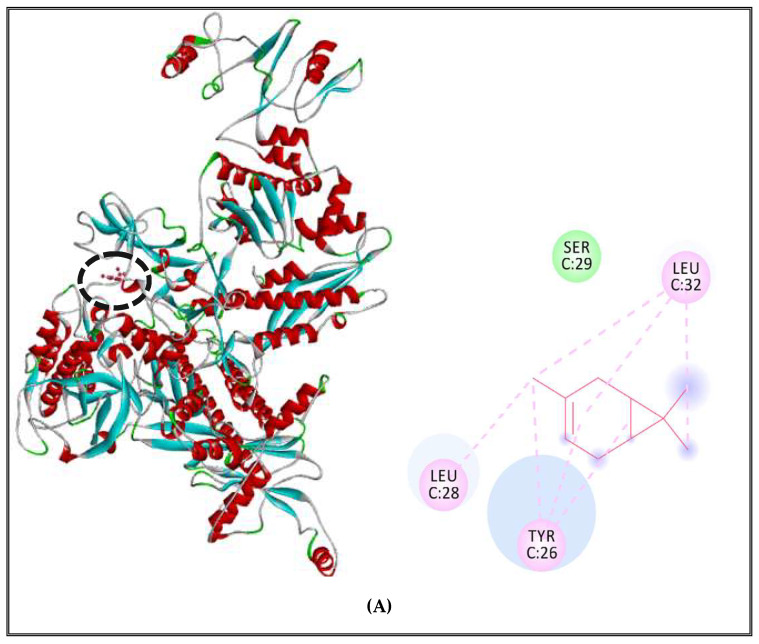

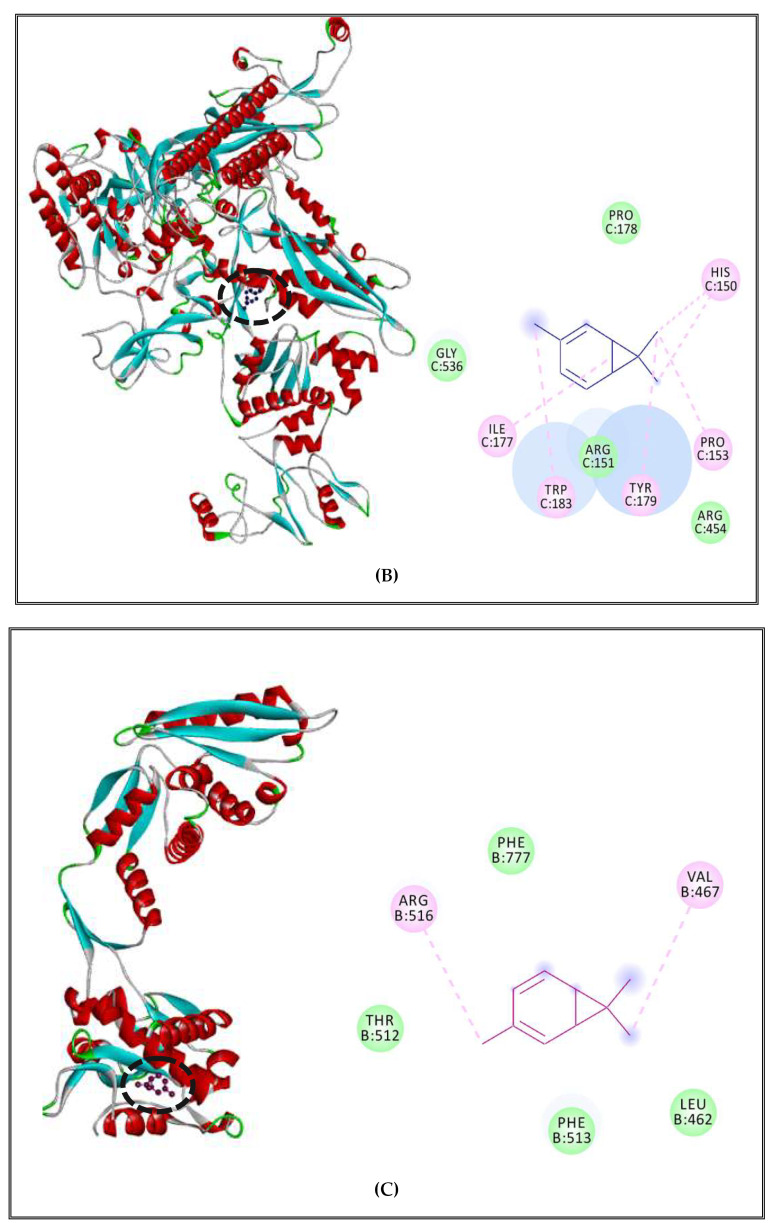
δ-3-carene complexed with DNA polymerase enzyme (**A**), RNA polymerase (**B**), topoisomerase II (**C**) of *S. enterica* Typhimurium.

On the other hand, Interaction profiles between δ-3-carene and Topoisomerase II, DNA and RNA polymerases of *S. aureus* and *S*. Typhimurium are outlined in Figure 6 and Figure 7). The complex between δ-3-carene and DNA polymerase of *S. aureus* displayed the existence of Alkyl interactions with ILE507, MET732, VAL722, ILE718, and Van der Waals interactions with LYS728, THR545, GLY546, ASN721, and ARG503 (Figure 6A). Moreover, it showed interactions with RNA polymerase via alkyl and Pi-alkyl interactions with VAL536, TRP39, and Ver der Waals with GLU413, GLU538, GLY540, ASN537, SER410, and SER36 (Figure 6B). Concerning topoisomerase II of *S. aureus*, δ-3-carene interacted with the receptor via alkyl interactions with VAL189, ALA640, and one Van der Waals interactions with ASN636, GLU193, ARG217, TYR192, TYR190, ASN191, TYR639, and VAL638 (Figure 6C).

DNA polymerase receptor of *S. enterica* Typhimurium complexed with δ-3-carene showed alkyl and Pi-alkyl interactions with LEU28, TYR26, LEU32 and only one Van der Waals interaction with SER29 (Figure 7A). Moreover, when complexed with RNA polymerase of S. Typhimurium, it revealed the existence of alkyl and Pi-alkyl interactions with ILE177, TRP183, TYR179, PRO153, HIS150 and Van der Waals with GLY536, ARG151, ARG454 and PRO178 (Figure 7B). Finally, the complex between δ-3-carene and the topoisomerase II of *Salmonella* exhibited the presence of alkyl interactions with VAL467, ARG516 and Van der Waals interactions with PHE777, LEU462, PHE513 and THR512 (Figure 7C).

**Figure 8:**
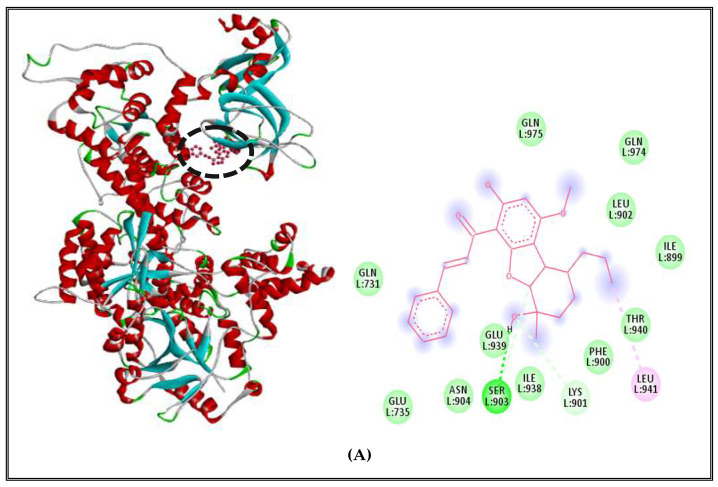

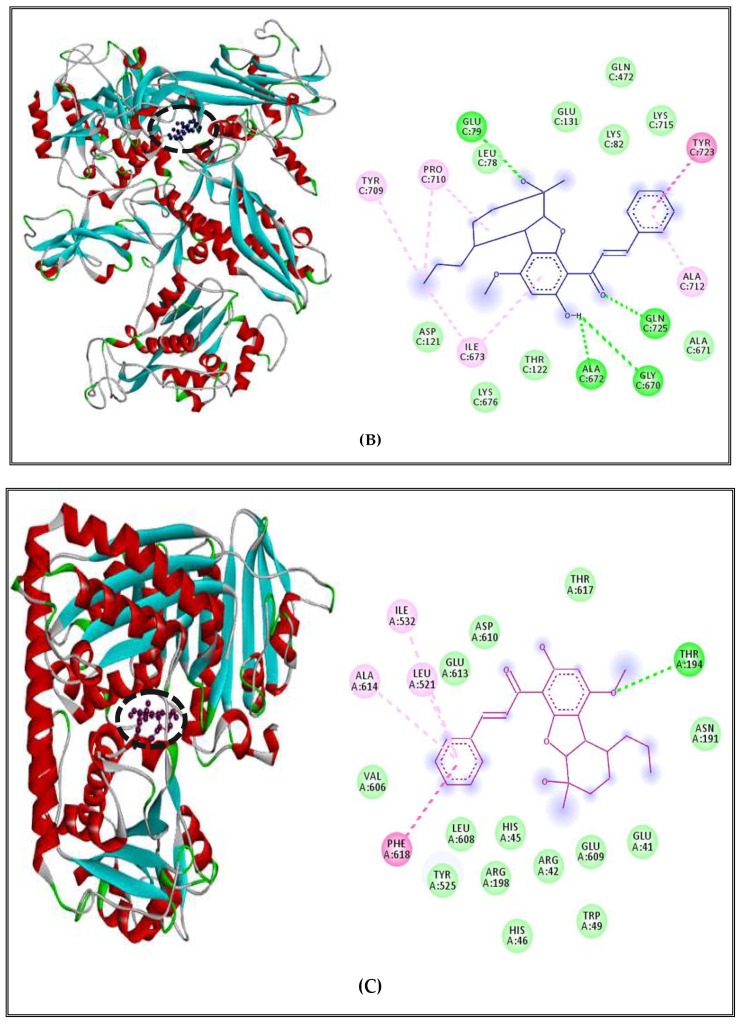
Borneol complexed with DNA polymerase enzyme (**A**), RNA polymerase (**B**), topoisomerase II (**C**) of *S.aureus*.

Results also indicated that DNA polymerase of *S. aureus* receptors complexed with borneol, showing 3 types of interactions including alkyl-type interaction with nonpolar amino acid LEU941, Van der Walls interactions with polar amino acids GLN731, GLN975, GLN974, THR940, GLU939, ASN904, GLU735 and nonpolar amino acids LEU902, ILE899,PHE900, ILE938, and one conventional hydrogen bond with polar amino acid SER903 (Figure 8A). On the other hand, the complex with RNA polymerase presented 4 types of interactions including Alkyl and Pi-alkyl interactions with a polar amino acid TYR709 and nonpolar amino acids PRO710, ILE673, ALA712, Van der Walls interactions with nonpolar amino acid LEU78, ALA671 and polar amino acids GLN131, GLN472, LYS82, LYS715, THR122, LYS676, ASP121 and a conventional hydrogen bonds with two polar amino acids GLU79, GLN725 and two nonpolar amino acids GLY670 and ALA672 (Figure 8B). Additionally, the complex between borneol and topoisomerase II of *S. aureus* indicated the presence of 4 different interactions, including Pi-alkyl interactions with nonpolar amino acids ILE532, Leu521, ALA614; Van der Walls interactions with polar amino acids GLU613, ASP610, THR617, ASN171, GLU41, GLU609, ARG42, HIS46, HIS45, ARG198, TYR525 and nonpolar amino acids TRP49, LEU608, VAL606; one conventional hydrogen bond with a polar amino acid THR194; and Pi–Pi interaction with nonpolar aromatic amino acid PHE618 (Figure 8C).

**Figure 9:**
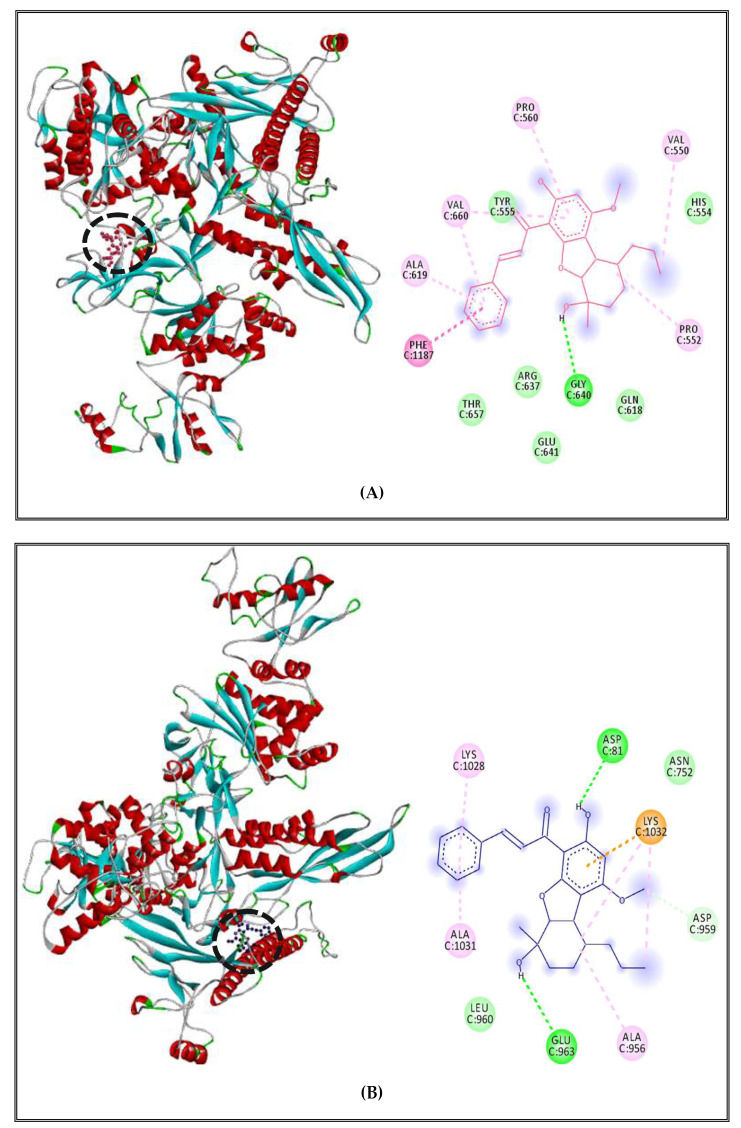

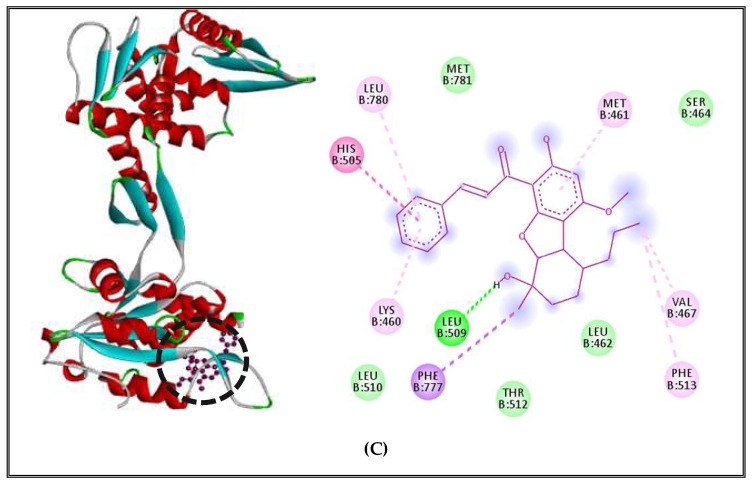
Borneol complexed with DNA polymerase enzyme (**A**), RNA polymerase (**B**), topoisomerase II (**C**) of *S. enterica* Typhimurium.

Borneol complex with *S. enterica* Typhimurium DNA polymerase showed different interactions with active site amino acids and the ligand: alkyl and Pi-alkyl interactions with nonpolar amino acids PRO552, PRO560, VAL550, VAL660, ALA619; Van der Walls interactions with polar amino acids such as TYR555, HIS554, GLN618, GLU641, ARG637, THR657; and only one conventional hydrogen bond with nonpolar amino acid GLY640 (Figure 9A). For the RNA polymerase, we found alkyl interactions with nonpolar amino acids ALA956, ALA1031 and a polar amino acid LYS1028; Van der Walls interactions with nonpolar amino acid LEU960 and polar amino acid ASN752, Pi-cation with polar amino acid LYS1032 and two conventional hydrogen bond with polar amino acid ASP81 and GLU963 (Figure 9B). Finally, the complex between borneol and topoisomerase II showed 5 types of interactions, including alkyl and Pi-alkyl with nonpolar amino acids such as LEU780, MET461, VAL467, PHE513, and a polar amino acid LYS460; Van der Walls interactions with nonpolar amino acids MET781, LEU462, LEU510 and polar amino acids such as SER464, THR512; one conventional hydrogen bond with nonpolar amino acid LEU509 and Pi-sigma interaction with nonpolar aromatic amino acid PHE777 (Figure 9C).

## 3. Materials and Methods

### 3.1. Plant Material, Essential Oil Extraction, and Gas Chromatography–Mass Spectrometry (GC–MS) Analysis

#### 3.1.1. Plant Material

Aerial parts of *C. sempervirens* were collected from Sfax, Tunisia (N: 34.4426°, E: 10.4537°) which is characterized by semi-arid climatic conditions. Aerial parts were harvested at the vegetative stage and were air-dried in obscurity at room temperature.

#### 3.1.2. Extraction and Analysis of (CSEO)

The EO of dried samples of *C. sempervirens* aerial parts was hydrodistilled for 3 h by using a Clevenger apparatus. The obtained (CSEO) was collected and dried over anhydrous sodium sulfate and maintained at 4 °C until analysis.

The analysis of the (CSEO) was carried out on a GC/MS HP model 6980 inert MSD, equipped with an Agilent Technologies capillary HP-5MS column (60 m × 0.25 mm, 0.25 mm film thickness) and coupled to a mass selective detector (MSD5973, ionization voltage 70 eV, all Agilent, Santa Clara, CA, USA). The carrier gas was helium and was used at 1.2 mL/min flow rate. The oven temperature program was as follows: 1 min at 100 °C ramped from 100 to 280 °C at 5 °C/min and 25 min at 280 °C. The chromatograph was equipped with a split/split less injector used in the split less mode. Identification of components was appointed by matching their mass spectra with Wiley Registry of Mass Spectral Data 7th edition (Agilent Technologies) and National Institute of Standards and Technology 05 MS (NIST) library data.

### 3.2. Antibacterial Activity

#### 3.2.1. Microorganisms and Growth Conditions

For antibacterial activity determination, bacteria used as indicator cells were obtained from international culture collections (ATCC); two Gram-positive bacteria: *Staphylococcus aureus* (*S. aureus*) ATCC 6538 and *Listeria monocytogenes* (*L*. *monocytogenes*) ATCC 19117, and two Gram-negative bacteria: *Salmonella enterica* Typhimurium ATCC 14028 and *Escherichia coli* (*E. coli*) ATCC 8739. According to the previous work of [81], the bacterial cultures were performed in Luria-Bertani (LB) agar medium composed of (g/L): peptone, 10; yeast extract, 5; NaCl, 5; and agar, 20 at pH 7.2, then the bacterial strains were incubated at 37 °C. Bacterial cultures were prepared by inoculating a loopful of each test bacteria in 3 mL of LB broth.

#### 3.2.2. Agar Diffusion Method

Antimicrobial activity of the essential oil of *Cupressus sempervirens* plant (CSEO) was evaluated by agar-well diffusion assay according to [82]. Fifteen milliliters of the molten agar (45 °C) were poured into sterile Petri dishes (Ø 90 mm). Working cell suspensions were prepared and 100 μL were evenly deposited onto the surface of plates containing LB agar medium. Plates were aseptically dried and then 5 mm wells were punched into the agar with a sterile Pasteur pipette. The (CSEO) was dissolved in dimethylsulfoxide (DMSO)/water (1/9; *v*/*v*) to a final concentration of 1 mg/mL and then filtered through 0.22 μm pore-size black polycarbonate filters. 100 μL of this filtered solution were placed into the wells and the plates were incubated at 37 °C.

#### 3.2.3. Minimal Inhibitory Concentration (MIC)

MIC of the (CSEO) against the four tested bacteria was determined using the micro-dilution method with serial dilution described by Chandrasekaran and Venkatesalu (2004) [83]. The final volume in each tube was 100 μL. The cell suspension was added to each test, to the final inoculum concentration of 10^6^ CF/mL of the corresponding indicator bacterium. The contents of the tubes were mixed by pipetting and were incubated for 24 h at 37 °C. The MIC was defined as the lowest concentration that inhibits the visible growth of the used indicator microorganism.

For the antibacterial activity determination (inhibition zones and CMIs), each experiment was carried out simultaneously three times under same conditions. The obtained diameters of inhibition zones reported in mm and the MIC values reported in mg/mL were quite similar and the reported results are the average of the two experiments.

### 3.3. Chemo-Computational Toxicity Evaluation Using In Silico Tools

#### 3.3.1. Toxicity Prediction of Compounds by VEGA HUB Software Using QSAR Method

The compounds from (CSEO): five recommended food preservatives (citric acid, BHA, L-ascorbic acid, propionic acid, and benzoic acid [84], rifamycin, and ciprofloxacin) were selected previously for molecular docking, as positive controls to inhibit DNA polymerase, RNA polymerase [85], and topoisomerase II [86] and efflux pumps inhibitors (cathinone and thioridazine) which are usually employed as controls to tackle multidrug-resistance to several antibiotics, were selected respectively. All compounds were subjected to 9 toxicity tests/measurements, including the genotoxicity/mutagenicity Caesar hybrid model for bacterial reverse mutation (Ames test), which consists of the detection of substances that could cause genetic mutations [87], the carcinogenicity CAESAR model for carcinogenicity which is based on spotting the ability of a molecule to induce tumors depends on its molecular structure [88]. The developmental toxicity model (CAESAR) and developmental/reproductive toxicity library (PG) help us to specify if the query compounds could be developmental toxicants and cause reproductive problems or not [89]. Besides, we selected another toxicity endpoint that facilitated the detection of endocrine-disrupting chemicals that interfere with the biosynthesis, metabolism, or action of endogenous hormones by the activation of their receptors. In this case, 3 models were employed: the estrogen receptor relative binding affinity model using 17-estradiol as the androgen receptor-mediated effect and thyroid receptor alpha and beta effects [90,91]. Finally, we used in vitro micronucleus activity model to evaluate the ability of an agent to cause DNA damage as an alteration in the structure or information content of genetic material in cells [92]. All toxicity endpoints measurement were performed by VEGA software version 1.1.5 using the QSAR (quantitative structure–activity relationship) approach [93].

#### 3.3.2. Rodent Oral Toxicity and Cytotoxicity of Selected Compounds Predicted by PROTOX II

PROTOX II is an in silico tool (Charite University of Medicine, Institute for Physiology, Structural Bioinformatics Group, Berlin, Germany) which is generally employed to evaluate multiple types of toxicity, such as acute toxicity, hepatotoxicity, cytotoxicity, carcinogenicity, mutagenicity, immunotoxicity, different toxicological pathways and targets, according to preliminary saved data obtained from both in vitro and in vivo assays [94].

In this research paper, PROTOX II was employed to predict rodent oral toxicity and cytotoxicity of *C. sempervirens* EO compounds, FDA approved drugs, food preservatives and EPIs in order to classify those compounds into several classes of toxicity using a globally harmonized system (GHS) of chemical labeling classification [95]. The SMILES (simplified molecular input line entry systems) of these compounds were introduced into the software for more chemo-computational toxicology evaluations.

### 3.4. In Vivo Toxicity Assessment Using Zebrafish Model 

#### 3.4.1. Zebrafish Maintenance and Embryos’ Collection

*Danio rerio*, a tropical freshwater fish, was used as a test species at the Laboratory of Molecular and Cellular Screening Processes (LPCMC), Center of Biotechnology of Sfax (CBS, Tunisia). Adults were maintained in culture under controlled conditions in a custom-made flow-through system. Each breeding group consisted of 7 females and 7 males, which were kept in 13 L circulation tanks at 26 ± 1 °C under continuous aeration. 14:10 h photoperiod cycle (light:dark) was maintained. Adult fish were fed twice a day with a commercially available dry food and fresh Artemia larvae.

The eggs were collected after a period of one hour of natural mating of 6 adult fish in a female/male ratio of 2:1. Then the eggs were washed thoroughly and rinsed several times with water. Healthy and developing embryos were selected within 1 hpf for exposure testing using a Stemi 2000-C stereomicroscope (Zeiss, Göttingen, Germany), transferred to crystallizing dishes and briefly stored in an incubator (26 ± 1 °C) until exposure. Screening the eggs before assaying ensured the exclusion of unfertilized eggs, injured, or deformed embryos. All experiments were approved by the Animal Ethics Committee of National School of Veterinary Medicine, IACUC, ENMV- Sidi Thabet, Tunisia (Permit No. CEEA-ENMV 44/22, 1 February 2022).

#### 3.4.2. Zebrafish Embryonic Toxicity Test and Determination of LC_50_

Embryotoxicity measurement in the zebrafish was elaborated by evaluating the mortality rate of the zebrafish embryos. Exposure of the embryos to the EO was performed based on the OECD Guideline for Testing of Chemicals 236—Acute Fish Embryo Toxicity Test (FET) [96]. Generally, for each concentration treatment, 20 fertilized eggs (at 1hpf) were used and placed in individual wells of 12-well plates.

The embryos were exposed to multiple concentrations of (CSEO) containing 0.1% dimethyl sulfoxide (DMSO) diluted in 2 mL of embryo water (embryonic medium). EO was serially diluted to produce 12 increasing concentrations (0.25; 0.5; 1; 2; 2.5; 3; 3.5; 4; 5; 6; 7; 8 μg/mL). The control (untreated group) was exposed only to 2 mL of embryo water. After treatment, the embryos were placed into at 27 °C and they were continuously examined every 24 h, using a stereomicroscope (Zeiss, Göttingen, Germany). Coagulation, absence of hatching and /or heartbeats were used as criteria to differentiate viable embryos from dead ones [97]. Finally, in order to LC_50_ values for (CSEO), the number of dead embryos was calculated in each concentration. The experiment was triplicated.

### 3.5. Interaction Study between the (CSEO) Molecules and Bacterial Protein Targets by Molecular Docking

#### 3.5.1. Homology Modeling of the Proteins

We performed homology modeling of proteins from two different microorganisms (1): *Staphylococcus aureus* (strain Mu50/ATCC 700699) and (2): *Salmonella* Typhimurium (strain LT2/SGSC1412/ATCC 700720) using the Swiss model server [98,99]. We selected DNA polymerase (Uniprot ID: P63979), RNA polymerase (Uniprot ID: Q932F8), and topoisomerase II (DNA gyrase) (Uniprot ID: P66936) from *Staphylococcus aureus* (strain Mu50/ATCC 700699). Similarly, DNA polymerase (Uniprot ID: P14567), RNA polymerase (Uniprot ID: P06173) and topoisomerase II (DNA gyrase) (Uniprot ID: P0A213) from *Salmonella* Typhimurium (strain LT2/SGSC1412/ATCC 700720). We also selected the AcrB efflux pump protein of *S*. Typhimurium (PDB ID: 5FFZ) from the NCBI database. The FASTA sequences of proteins were obtained from the UNIPORT server [100,101] and submitted to the Swiss Model server for Automatic Homology Modeling using the default parameters. The finding of the best template proteins was performed by the BLASTp program [102]. The predicted homology models are ranked based on the target-template protein sequence identities, QMEAN Z score, and GMQE score.

#### 3.5.2. Validation of Protein Models

The obtained protein models from the Swiss model server were subjected to model validation using the Profunc Server [103]. The Profunc server has an integrated protein analysis tool known as Procheck, which analyzes the overall quality of the models using the Ramachanderan plot based on the distribution of dihedral angles of the amino acids backbone Phi (Φ) and Psi (Ψ) angles [104]. We also considered the QMEAN Z score and GMQE score from the Swiss model server for the selection of the best homology models [105].

#### 3.5.3. Binding Site Prediction

All selected protein models were subjected to binding site prediction using the Profunc server [103]. Our query protein structures are searched against the protein database with known binding site residues using “reverse template comparison vs. structure in PDB approach” integrated in the SiteSeer program. The binding site residues are extracted from best hits with E-value 0.

#### 3.5.4. Selection of the Compounds

Based on the available literature, two antibiotics, Rifamycin SV and Ciprofloxacin, were used as controls and were downloaded from the Drug Bank database [106]. Twenty-seven compounds of *C. sempervirens* and two efflux pump inhibitors (cathinone and thioridazine) were downloaded from the Pubchem database [107]. Their Smiles strings were obtained from the Pubchem database and converted into a 3D structure via the Corina server [108]. All files were saved in the pdb file format.

#### 3.5.5. Molecular Docking by Autodock Vina

The virtual screening of the compounds was performed against proteins using the Autodock Vina [109]. First, the compound pdbqt files were prepared by Autodock.4.2 software. All hydrogen atoms were added to the compound’s structures, followed by the merging of the nonpolar hydrogen atoms. Subsequently, Gasteiger charges were added to each atom. The number of rotatable bonds is set to be maximum according to the torsional bonds in the compound. All proteins files were also prepared by Autodock.4.2 software. Like compounds, all hydrogen atoms were added to the protein’s structures, followed by merging the nonpolar hydrogen atoms. Subsequently, Gasteiger charges were added to each atom and Kollman united atom charges were assigned to the receptor atom. The grid box was built around the binding sites of proteins by making a grid box size of 126 x 126 × 126 with a grid spacing of 0.375 Å. This grid box dimension covered the whole binding site and provided enough space for translation and rotation of ligands. The corresponding grid center coordinates were set according to the respective binding site residues of the proteins. The conFigure file (*conf*) of the Autodock vina was set with name of protein.pdbqt, information about *center_x, center_y, center_z* and box size of *xyz*. The exhaustivness value was set to 10 with number of modes (*num_modes*) of 200 and *energy_range* to 4. All autodock vina executable files were put in same folder, where the compounds and proteins files were present. We used open babel tool for converting all compounds pdb files in to pdbqt format using the command “obabel *.pdb -opdbqt -m”. We have developed our own docking script for automation of docking simulation by Autodock vina. All compound pdbqt files were added to the ligand.txt file for our docking perl script. Our perl script was set to take each ligand and screen against the binding site of the protein and all outputs were stored in the docking folder. The single docked conformation was selected from each docking round based on the clustering RMSD and lowest binding energy.

The most stable conformations of the ligand molecule were selected based on the lowest binding energy and their binding mode at the active site of proteins and analyzed by discovery studio software for h-bond analysis and non-bonded interactions between compounds and proteins [110].

### 3.6. Statistical Analysis

All tests were assayed in triplicate and expressed as the mean ± standard deviation of the measurements. The statistical program SPSS version 21.00 for Windows (SPSS Inc., Chicago, IL, USA) was used to analyze data. Variance was analyzed by one-way ANOVA and Tukey’s multiple range tests were calculated for the significant data at *p* < 0.05.

## 4. Conclusions

The tendency towards the application of EOs as safer antimicrobial agents has increased. Results of this study evidenced the anti-food-borne bacterial activities of (CSEO) against *S. enterica* Typhimurium and *S. aureus*. Interestingly, α-pinene, δ-3-carene, and borneol, belonging to monoterpenes, can increase the cell wall permeability and inhibit DNA and RNA polymerases and topoisomerase II of bacteria. Predicted molecular docking analysis showed that these three compounds were highly reactive molecules when compared to the reference antibiotics. These findings indicate that (CSEO) can be used in targeted drug development to combat antibiotic resistance associated with efflux pump expression, modulation of DNA topology, and DNA and RNA synthesis. In addition, mutagenic, toxicological, and carcinogenic properties of all (CSEO) compounds vs. some recommended food preservatives commonly used in the food industry were investigated by applying in silico tools and software. Notably, we revealed an absence of toxic effects of many (CSEO) components. Throughout the zebrafish model, we confirmed the safety of (CSEO). Overall, experimental validation by studying in vitro the process of impairment of membrane permeability and replication of pathogenic bacteria insured by (CSEO) would be required for conclusive confirmation in order to be applied to reduce the impact of the diseases caused by such pathogenic microorganisms.

## Figures and Tables

**Table 1:** Chemical composition of (CSEO).

Compound	Molar Mass (g/mol)	Molecular Formula	Retention Time (min)	EO (%)
**β-terpinene**	136.23	C_10_H_16_	4.80	0.11
**Tricyclene**	136.23	C_10_H_16_	5.25	0.22
**α-pinene**	136.23	C_10_H_16_	5.76	38.47
**α-fenchene**	136.23	C_10_H_16_	5.95	1.36
**Sabinene**	136.23	C_10_H_16_	6.67	1.18
**β-pinene**	136.23	C_10_H_16_	6.75	2.04
**β-myrcene**	136.23	C_10_H_16_	7.22	2.78
**δ-3-carene**	136.23	C_10_H_16_	7.91	25.14
**D-limonene**	136.23	C_10_H_16_	7.99	5.84
**P-cymene**	134.22	C_10_H_14_	8.23	0.86
**Linalool**	154.25	C_10_H_18_O	10.62	0.38
**Isopulegol**	154.25	C_10_H_18_O	11.96	0.88
**Citronellal**	154.25	C_10_H_18_O	12.23	5.33
**Borneol**	154.25	C_10_H_18_O	12.58	1.37
**Terpinen-4-ol**	154.25	C_10_H_18_O	12.92	1.55
**α-terpineol**	154.25	C_10_H_18_O	13.39	0.54
**β-citronellol**	156.26	C_10_H_20_O	14.54	0.21
**α-fenchyl acetate**	196.29	C_12_H_20_O_2_	15.95	1.28
**Camphene**	136.23	C_10_H_16_	16.29	0.29
**α-terpinyl acetate**	196.29	C_12_H_20_O_2_	17.73	2.82
**α-zingibirene**	204.35	C_15_H_24_	19.31	0.52
**α-carophyllene**	204.35	C_15_H_24_	19.49	0.83
**α-humulene**	204.35	C_15_H_24_	20.36	0.18
**Germacrene D**	204.35	C_15_H_24_	20.60	0.83
**α-amorphene**	204.35	C_15_H_24_	20.94	0.18
**δ-cadinene**	204.35	C_15_H_24_	20.09	0.30
**Cedrol**	222.37	C_15_H_26_O	24.02	2.24
**Monoterpenes hydrocarbons**				**78.29 (%)**
**Oxygenated monoterpenes**				**17.28 (%)**
**Sesquiterpens**				**2.84 (%)**
**Total**				**98.41 (%)**

**Table 2:** Antibacterial activity of (CSEO); zones of growth inhibition expressed in (mm) and minimum inhibitory concentrations (MICs) expressed in (µg/mL) of the (CSEO) and the standard antibiotic gentamicin. All tests were performed in triplicate; values with a different letter (a,b) within a row for each antibacterial test are significantly different (*p* < 0.05).

Bacterial Strains	Inhibition Zones Diameters (mm)		MIC (µg/mL)	
	(CSEO)	Gentamicin	(CSEO)	Gentamicin
*S. aureus* ATCC 6538	21 ± 1.00 ^a^	20 ± 0.83 ^a^	6.25 ± 0.00 ^a^	12.5 ± 0.00 ^b^
*L. monocytogenes* ATCCC 19117	21 ± 0.83 ^a^	20 ± 0.66 ^a^	6.25 ± 0.00 ^a^	12.5 ± 0.00 ^b^
*S.* Typhimurium ATCC 14028	15 ± 0.5 ^a^	25 ± 1.00 ^b^	12.5 ± 0.00 ^b^	2.5 ± 0.00 ^a^
*E. coli* ATCC 8739	15 ± 0.5 ^a^	25 ± 1.25 ^b^	12.5 ± 0.00 ^b^	2.5 ± 0.00 ^a^

**Table 3:** Toxicity predictions of (CSEO) compounds and selected antibiotics using Vega QSAR model.

Toxicity Measurements	Mutagenicity (Ames Test) Model (CAESAR) 2.1.13	Carcinogenicity Model (CAESAR) 2.1.9	Developmental Toxicity Model (CAESAR) 2.1.7	Developmental/Reproductive Toxicity Library (PG) 1.1.0	Estrogen Receptor Relative Binding Affinity Model (IRFMN)	Androgen Receptor-Mediated Effect (IRFMN/COMPARA) 1.0.0	Thyroid Receptor Alpha Effect (NRMEA) 1.0.0	Thyroid Receptor Beta Effect (NRMEA) 1.0.0	In Vitro Micronucleus Activity (IRFMN/VERMEER) 1.0.0
Compound									
**β-terpinene**	-	-	-	-	-	-	-	-	-
**Tricyclene**	-	-	-	-	+	-	-	-	-
**α-pinene**	-	-	+	-	-	-	-	-	-
**α-fenchene**	-	-	-	-	-	-	-	-	-
**Sabinene**	-	-	-	-	-	-	-	-	-
**β-pinene**	-	-	+	-	-	-	-	-	-
**β-myrcene**	-	+	-	-	-	-	-	-	+
**δ-3-carene**	-	-	-	-	-	-	-	-	-
**D-limonene**	-	+	-	-	-	-	-	-	-
**P-cymene**	-	-	-	+	-	-	-	-	-
**Linalool**	-	-	-	-	-	-	-	-	+
**Isopulegol**	-	+	+	+	-	-	-	-	-
**Citronellal**	-	-	-	-	-	-	-	-	-
**Borneol**	-	-	-	-	-	-	-	-	-
**Terpinen-4-ol**	-	-	+	-	-	-	-	-	-
**α-terpineol**	-	-	+	-	-	-	-	-	-
**β-citronellol**	-	-	-	-	-	-	-	-	-
**α-fenchyl acetate**	-	-	-	-	-	-	-	-	-
**Camphene**	-	-	-	-	-	-	-	-	-
**α-terpinyl acetate**	-	-	-	-	-	-	-	-	-
**α-zingibirene**	-	-	-	-	-	-	-	-	+
**α-carophyllene**	-	-	-	-	-	-	-	-	+
**α-humulene**	-	-	-	-	-	-	-	-	+
**Germacrene D**	-	-	-	-	-	-	-	-	+
**α-amorphene**	-	+	+	-	-	-	-	-	+
**δ-cadinene**	-	+	+	-	-	-	-	-	-
**Cedrol**	-	-	+	-	-	-	-	-	-
**Ciprofloxacin**	+	-	+	+	-	-	-	-	+
**Rifamycin SV**	+	-	+	-	-	-	-	-	+

(**-**): Non toxicant/inactive; (**+**): Toxicant/active.

**Table 4:** Toxicity assessment of food preservatives and efflux pumps inhibitors (controls) using the Vega QSAR model.

Toxicity Measurements	Citric Acid	Butylated Hydroxyanisol (BHA)	Ascorbic Acid	Propionic Acid	Benzoic Acid	Cathinone	Thioridazine
**Mutagenicity (Ames test) model (CAESAR) 2.1.13**	-	-	-	-	-	-	-
**Carcinogenicity model (CAESAR) 2.1.9**	-	+	-	-	-	-	-
**Developmental Toxicity model (CAESAR) 2.1.7**	-	-	-	+	+	+	+
**Developmental/Reproductive Toxicity library (PG) 1.1.0**	-	-	-	+	-	+	+
**Estrogen Receptor Relative Binding Affinity model (IRFMN)**	-	-	-	-	-	-	+
**Androgen Receptor-mediated effect (IRFMN/COMPARA) 1.0.0**	-	-	-	-	-	-	-
**Thyroid Receptor Alpha effect (NRMEA) 1.0.0**	-	-	-	-	-	-	-
**Thyroid Receptor Beta effect (NRMEA) 1.0.0**	-	-	-	-	-	-	-
**In vitro Micronucleus activity (IRFMN/VERMEER) 1.0.0**	+	-	+	**Not predicted**	-	+	+

(**-**): Non toxicant/inactive; (**+**): Toxicant/active.

**Table 5:** Rodent oral toxicity and cytotoxicity of (CSEO) compounds, food preservatives, and efflux pumps inhibitors (EPI) predicted by the PROTOX II tool.

Compound	Cytotoxicity	Probability	LD 50 (mg/kg)	Toxicity Class
**β-terpinene**	Inactive	0.80	4400	5
**Tricyclene**	Inactive	0.77	15,380	6
**α-pinene**	Inactive	0.75	3700	5
**α-fenchene**	Inactive	0.74	5000	5
**Sabinene**	Inactive	0.71	5000	5
**β-pinene**	Inactive	0.71	4700	5
**β-myrcene**	Inactive	0.75	5000	5
**δ-3-carene**	Inactive	0.71	4800	5
**D-limonene**	Inactive	0.82	4400	5
**P-cymene**	Inactive	0.89	3	1
**Linalool**	Inactive	0.82	2200	5
**Isopulegol**	Inactive	0.93	5000	5
**Citronellal**	Inactive	0.82	2420	5
**Borneol**	Inactive	0.88	500	4
**Terpinen-4-ol**	Inactive	0.88	1016	4
**α-terpineol**	Inactive	0.64	2830	5
**β-citronellol**	Inactive	0.86	3450	5
**α-fenchyl acetate**	Inactive	0.73	3100	5
**Camphene**	Inactive	0.76	5000	5
**α-terpinyl acetate**	Inactive	0.80	4800	5
**α-zingibirene**	Inactive	0.82	1680	4
**α-caryophyllene**	Inactive	0.79	3650	5
**α-humulene**	Inactive	0.79	3650	5
**Germacrene D**	Inactive	0.83	5300	5
**α-amorphene**	Inactive	0.76	4400	5
**δ-cadinene**	Inactive	0.69	4390	5
**Cedrol**	Inactive	0.87	2000	4
**Rifamycin SV**	Inactive	0.60	2120	5
**Ciprofloxacin**	Inactive	0.92	2000	4
**Citric acid**	Inactive	0.73	80	3
**BHA**	Inactive	0.83	700	4
**L-ascorbic acid**	Inactive	0.65	3367	5
**Propionic acid**	Inactive	0.75	300	3
**Benzoic acid**	Inactive	0.86	235	3
**Cathinone**	Inactive	0.82	400	4
**Thioridazine**	Inactive	0.68	360	4

Class 1: fatal if swallowed (LD50 ≤ 5); Class 2: fatal if swallowed (5 < LD50 ≤ 50); Class 3: toxic if swallowed (50 < LD50 ≤ 300); Class 4: harmful if swallowed (300 < LD50 ≤ 2000); Class 5: may be harmful if swallowed (2000 < LD50 ≤ 5000); Class 6: nontoxic (LD50 > 5000).

**Table 6:** Molecular docking results for complexes between compounds of (CSEO) and efflux pump targets of *S. aureus* and *S. enterica* Typhimurium by using Autodock Vina (kcal/mol).

Compound	AcrsB Efflux Pump	MepR
β-terpinene	−5.1	−5.5
Tricyclene	−5.8	−4.7
**α-pinene**	**−6.7**	**−6.5**
α-fenchene	−5.3	−4.8
Sabinene	−5.0	−5.2
β-pinene	−5.0	−4.9
β-myrcene	−4.8	−4.5
**δ-3-carene**	**−6.4**	**−6.2**
D-limonene	−5.3	−5.3
P-cymene	−6.2	−5.6
Linalool	−4.8	−4.4
Isopulegol	−5.2	−5.1
Citronellal	−4.2	−3.9
**Borneol**	**−7.9**	**−7.7**
Terpinen-4-ol	−5.3	−5.0
α-terpineol	−5.3	−5.3
β-citronellol	−4.4	−4.3
α-fenchyl acetate	−6.4	−4.3
Camphene	−5.4	−4.8
α-terpinyl acetate	−6.2	−5.2
α-zingibirene	−5.4	−5.6
α-carophyllene	−6.0	−6.3
α-humulene	−6.5	−6.3
Germacrene D	−7.0	−6.4
α-amorphene	−6.5	−6.6
δ-cadinene	−6.6	−6.7
Cedrol	−6.3	−6.5
**Cathinone**	−4.8	-
**Thioridazine**	-	−6.7

**Table 7:** Molecular docking results for complexes between compounds of *C. sempervirens* EO and protein targets of *S. aureus* and *S.* Typhimurium using Autodock Vina (kcal/mol).

	*S. aureus* (Strain Mu50/ATCC 700699)			*S.* Typhimurium (strain LT2/SGSC1412/ATCC 700720)		
Compound	DNA Polymerase	RNA Polymerase	Topoisomerase II	DNA Polymerase	RNA Polymerase	Topoisomerase II
**β-terpinene**	−5.2	−5.8	−5.5	−5.3	−6	−5.4
**Tricyclene**	−5.2	−5	−5.4	−5.2	−5	−5.2
**α-pinene**	−5.2	−5.1	−5.3	−5.4	−5.4	−5.2
**α-fenchene**	−5.4	−5.6	−5.2	−5.2	−5.1	−5.5
**Sabinene**	−5.2	−5.4	−5.4	−5.2	−5.2	−5.1
**β-pinene**	−5.5	−5.1	−5.3	−5.4	−5.4	−5.2
**β-myrcene**	−4.6	−4.3	−5.3	−4.7	−4.3	−4.3
**δ-3-carene**	−5.1	−6.1	−5.2	−5.6	−5.4	−5.6
**D-limonene**	−4.9	−4.6	−5.4	−4.9	−5.2	−5.1
**P-cymene**	−5.4	−5.2	−5.6	−5.4	−5.5	−5.2
**Linalool**	−4.8	−4.8	−5.2	−5.1	−4.5	−4.2
**Isopulegol**	−5.3	−5.8	−5.2	−5.4	−5.4	−5
**Citronellal**	−4.5	−4.4	−4.8	−4.6	−4.8	−4.2
**Borneol**	−7.7	−8.2	−8.8	−7.7	−7.3	−7.4
**Terpinen-4-ol**	−5.2	−5.3	−5.7	−5.7	−5.7	−5.3
**α-terpineol**	−5.3	−5.9	−5.6	−5.7	−5.7	−5.2
**β-citronellol**	−4.8	−4.3	−5	−5.1	−4.6	−4.7
**α-fenchyl acetate**	−5.7	−5.4	−5.6	−5.2	−5.3	−5.1
**Camphene**	−5.2	−5.4	−5.1	−5.5	−5.5	−5.2
**α-terpinyl acetate**	−5.4	−5.1	−6.4	−6	−6	−5.3
**α-zingibirene**	−5.8	−5.1	−6.1	−6.1	−5.2	−5.8
**α-carophyllene**	−6.2	−5.9	−6.8	−6.1	−6.1	−6.3
**α-humulene**	−6.1	−6.3	−6.8	−6.1	−6.1	−6.3
**Germacrene D**	−6.5	−7.1	−6.7	−6.2	−6.2	−6.5
**α-amorphene**	−6.8	−6.2	−6.9	−6.8	−6.2	−6
**δ-cadinene**	−6.3	−6.7	−6.7	−6.5	−6.7	−6.1
**Cedrol**	−6.6	−6.7	−6.8	−6.9	−6.2	−6.7
**Rifamycin SV**	−9.2	−9.8	-	−8.4	−8.6	-
**Ciprofloxacin**	-	-	−6.7	-	-	−6.3

**Table 8:** Interaction details of (CSEO) compounds and the active sites of selected bacterial targets.

Bacteria	Compound	Targets	Number of Residues Interacting	Residues with H-Bond
*S. aureus* (strain Mu50/ATCC 700699)	α-pinene	DNA polymerase	6	-
		RNA polymerase	6	-
		Topoisomerase II	6	-
	δ-3-carene	DNA polymerase	8	-
		RNA polymerase	4	-
		Topoisomerase II	4	-
	Borneol	DNA polymerase	4	Ser 901, Lys 901
		RNA polymerase	11	Glu 79, Ala 672, Gly 670, Gln 725
		Topoisomerase II	5	Thr 194
*S.* Typhimurium (strain LT2/SGSC1412/ATCC 700720)	α-pinene	DNA polymerase	3	-
		RNA polymerase	9	-
		Topoisomerase II	8	-
	δ-3-carene	DNA polymerase	7	-
		RNA polymerase	6	-
		Topoisomerase II	2	-
	Borneol	DNA polymerase	8	Gly 640
		RNA polymerase	8	Asp81, Glu 963
		Topoisomerase II	8	Leu 509

## Data Availability

All data generated or analyzed during this study are included in this published article.

## Sample Availability

Samples of the compounds are not available from the authors.

## Appendix A

**Table A1:** Receptors models of the two pathogenic bacteria used to analyze the molecular docking with the major constituents of the (CSEO).

Bacterial Strain	Bacterial Target	Receptor (UniprotKB)	Template	Identity (%)	Ramachandran Plot		QMEAN
					Favoured Regions (%)	Additional Allowed Regions (%)	
***Staphylococcus aureus* (strain Mu50/ATCC 700699)**	DNA polymerase	P63979	4IQJ.1.L	34.77	87.9	10.1	−3.26
	RNA polymerase	Q932F8	6WVK.1.C	81.09	85.5	12.7	−2.36
	Topoisomerase II	P66936	6GAV.1.A	54.42	88.4	10.7	−1.69
***Salmonella* Typhimurium (strain LT2/SGSC1412/ATCC 700720)**	DNA polymerase	P14567	5FKU.1.A	96.72	88.0	10.0	−2.35
	RNA polymerase	P06173	4LLG.2.C	98.66	88.0	11.0	−1.11
	Topoisomerase II	P0A213	4TMA.2.B	95.41	90.2	9.2	−1.82
